# Supplementary material for: Smooth Interpolating Curves with Local Control and Monotone Alternating Curvature
Source: Comput Graph Forum. 2022 Oct 6;41(5):25–38. doi: 10.1111/cgf.14600 (PMC9827861; doi:10.1111/cgf.14600)
Supplement: Supplementary file 1 — Supplement Material [file CGF-41-25-s001.zip › Local-Smooth-Interpolating-MonoCurvature/extern/clothoids/submodules/Utils/docs/api-cpp/program_listing_file_CPUinfo.cc.html]

Program Listing for File CPUinfo.cc — Utils v1.2

### Navigation

- index
- toc
- Utils »
- Program Listing for File CPUinfo.cc

# Program Listing for File CPUinfo.cc¶

↰ Return to documentation for file (`CPUinfo.cc`)

```
/*--------------------------------------------------------------------------*\
 |                                                                          |
 |  Copyright (C) 2011                                                      |
 |                                                                          |
 |         , __                 , __                                        |
 |        /|/  \               /|/  \                                       |
 |         | __/ _   ,_         | __/ _   ,_                                |
 |         |   \|/  /  |  |   | |   \|/  /  |  |   |                        |
 |         |(__/|__/   |_/ \_/|/|(__/|__/   |_/ \_/|/                       |
 |                           /|                   /|                        |
 |                           \|                   \|                        |
 |                                                                          |
 |      Enrico Bertolazzi                                                   |
 |      Dipartimento di Ingegneria Industriale                              |
 |      Universita` degli Studi di Trento                                   |
 |      email: enrico.bertolazzi@unitn.it                                   |
 |                                                                          |
\*--------------------------------------------------------------------------*/

/*
SIMD class to detect presence of SIMD version 1.2.0

Copyright (c) 2008 Wong Shao Voon
1.1.0 - Contains the 3DNow, 3DNow+ and MMX+ detection bug fix by Leonardo Tazzini
1.2.0 - Added AMD's SSE4a and SSE5

The Code Project Open License (CPOL)
http://www.codeproject.com/info/cpol10.aspx
*/

#ifndef DOXYGEN_SHOULD_SKIP_THIS

#include "Utils.hh"
#include "Utils_CPU_info.hh"
#include <map>


#ifdef __ARM_ARCH

namespace Utils {

  #ifndef DOXYGEN_SHOULD_SKIP_THIS
  using std::string;
  using std::map;
  #endif

  void
  info( bool & m_bMMX,
        bool & m_bMMXplus,
        bool & m_bSSE,
        bool & m_bSSE2,
        bool & m_bSSE3,
        bool & m_bSSSE3,
        bool & m_bSSE41,
        bool & m_bSSE42,
        bool & m_bSSE4a,
        bool & m_bSSE5,
        bool & m_b3Dnow,
        bool & m_b3DnowExt ) {

    m_bMMX      = false;
    m_bMMXplus  = false;
    m_bSSE      = false;
    m_bSSE2     = false;
    m_bSSE3     = false;
    m_bSSSE3    = false;
    m_bSSE41    = false;
    m_bSSE42    = false;
    m_bSSE4a    = false;
    m_bSSE5     = false;
    m_b3Dnow    = false;
    m_b3DnowExt = false;
  }

  bool has_MMX()      { return false; }
  bool has_MMXplus()  { return false; }
  bool has_SSE()      { return false; }
  bool has_SSE2()     { return false; }
  bool has_SSE3()     { return false; }
  bool has_SSSE3()    { return false; }
  bool has_SSE41()    { return false; }
  bool has_SSE42()    { return false; }
  bool has_SSE4a()    { return false; }
  bool has_SSE5()     { return false; }
  bool has_3Dnow()    { return false; }
  bool has_3DnowExt() { return false; }

  string
  cpuInfo() {
    return "ARM-unsupported";
  }

}

#else

#define EDX_MMX_bit      0x800000UL   // 23 bit
#define EDX_SSE_bit      0x2000000UL  // 25 bit
#define EDX_SSE2_bit     0x4000000UL  // 26 bit
#define EDX_3DnowExt_bit 0x40000000UL // 30 bit
#define EDX_3Dnow_bit    0x80000000UL // 31 bit
#define EDX_MMXplus_bit  0x400000UL   // 22 bit

#define ECX_SSE3_bit     0x1UL        //  0 bit
#define ECX_SSSE3_bit    0x200UL      //  9 bit
#define ECX_SSE41_bit    0x80000UL    // 19 bit
#define ECX_SSE42_bit    0x100000UL   // 20 bit

#define ECX_SSE4A_bit    0x40UL       //  6 bit
#define ECX_SSE5_bit     0x800UL      // 11 bit

namespace Utils {

  #ifndef DOXYGEN_SHOULD_SKIP_THIS
  using std::string;
  using std::map;
  #endif

  #if defined(UTILS_OS_WINDOWS)
    #include <intrin.h>
    void
    cpuId( unsigned long CPUInfo[4], unsigned long l ) {
      int CPUInfo1[4];
      __cpuid( CPUInfo1, l );
      CPUInfo[0] = CPUInfo1[0];
      CPUInfo[1] = CPUInfo1[1];
      CPUInfo[2] = CPUInfo1[2];
      CPUInfo[3] = CPUInfo1[3];
    }
  #else
    #if defined(__i386__) && defined(__PIC__)
      /* %ebx may be the PIC register.  */
      #if __GNUC__ >= 3
        #define __cpuid(level, a, b, c, d)                        \
          __asm__ ( "xchg{l}\t{%%}ebx, %1\n\t"                    \
                    "cpuid\n\t"                                       \
                    "xchg{l}\t{%%}ebx, %1\n\t"                    \
                    : "=a" (a), "=r" (b), "=c" (c), "=d" (d)    \
                    : "0" (level))
      #else
        /* Host GCCs older than 3.0 weren't supporting Intel asm syntax
           nor alternatives in i386 code.  */
        #define __cpuid(level, a, b, c, d)                        \
          __asm__ ( "xchgl\t%%ebx, %1\n\t"                        \
                    "cpuid\n\t"                                       \
                    "xchgl\t%%ebx, %1\n\t"                        \
                    : "=a" (a), "=r" (b), "=c" (c), "=d" (d)    \
                    : "0" (level))
      #endif
    #else
      #define __cpuid(level, a, b, c, d)                        \
        __asm__ ( "cpuid\n\t"                                       \
                  : "=a" (a), "=b" (b), "=c" (c), "=d" (d)  \
                  : "0" (level))
    #endif
    void
    cpuId( unsigned long CPUInfo[4], unsigned long l ) {
      __cpuid( l, CPUInfo[0], CPUInfo[1], CPUInfo[2], CPUInfo[3] );
    }
  #endif

  static
  void
  info(
    unsigned long   CPUInfo[4],
    unsigned long   CPUInfoExt[4],
    unsigned long & ECX,
    unsigned long & EDX
  ) {

    ECX = EDX = CPUInfoExt[0] = CPUInfoExt[1] = CPUInfoExt[2] = CPUInfoExt[3] = 0;

    cpuId(CPUInfo, 0);

    if( CPUInfo[0] >= 1 ) {
      cpuId(CPUInfo, 1);
      ECX = CPUInfo[2];
      EDX = CPUInfo[3];
    }

    cpuId(CPUInfo, 0x80000000UL);
    if( CPUInfo[0] >= 0x80000001UL ) cpuId(CPUInfoExt, 0x80000001UL);
  }

  void
  info(
    bool & m_bMMX,
    bool & m_bMMXplus,
    bool & m_bSSE,
    bool & m_bSSE2,
    bool & m_bSSE3,
    bool & m_bSSSE3,
    bool & m_bSSE41,
    bool & m_bSSE42,
    bool & m_bSSE4a,
    bool & m_bSSE5,
    bool & m_b3Dnow,
    bool & m_b3DnowExt
  ) {

    m_bMMX      = false;
    m_bMMXplus  = false;
    m_bSSE      = false;
    m_bSSE2     = false;
    m_bSSE3     = false;
    m_bSSSE3    = false;
    m_bSSE41    = false;
    m_bSSE42    = false;
    m_bSSE4a    = false;
    m_bSSE5     = false;
    m_b3Dnow    = false;
    m_b3DnowExt = false;

    unsigned long ECX, EDX, CPUInfo[4], CPUInfoExt[4];
    info( CPUInfo, CPUInfoExt, ECX, EDX );

    if ( EDX_MMX_bit      & EDX           ) m_bMMX      = true;
    if ( EDX_SSE_bit      & EDX           ) m_bSSE      = true;
    if ( EDX_SSE2_bit     & EDX           ) m_bSSE2     = true;
    if ( ECX_SSE3_bit     & ECX           ) m_bSSE3     = true;
    if ( ECX_SSSE3_bit    & ECX           ) m_bSSSE3    = true;
    if ( ECX_SSE41_bit    & ECX           ) m_bSSE41    = true;
    if ( ECX_SSE42_bit    & ECX           ) m_bSSE42    = true;
    if ( EDX_3DnowExt_bit & CPUInfoExt[3] ) m_b3DnowExt = true;
    if ( EDX_3Dnow_bit    & CPUInfoExt[3] ) m_b3Dnow    = true;
    if ( EDX_MMXplus_bit  & CPUInfoExt[3] ) m_bMMXplus  = true;
    if ( ECX_SSE4A_bit    & CPUInfoExt[2] ) m_bSSE4a    = true;
    if ( ECX_SSE5_bit     & CPUInfoExt[2] ) m_bSSE5     = true;
  }

  bool
  has_MMX() {
    unsigned long ECX, EDX, CPUInfo[4], CPUInfoExt[4];
    info( CPUInfo, CPUInfoExt, ECX, EDX );
    return (EDX_MMX_bit & EDX) != 0;
  }

  bool
  has_MMXplus() {
    unsigned long ECX, EDX, CPUInfo[4], CPUInfoExt[4];
    info( CPUInfo, CPUInfoExt, ECX, EDX );
    return (EDX_SSE_bit & EDX) != 0;
  }

  bool
  has_SSE() {
    unsigned long ECX, EDX, CPUInfo[4], CPUInfoExt[4];
    info( CPUInfo, CPUInfoExt, ECX, EDX );
    return (EDX_SSE_bit & EDX) != 0;
  }

  bool
  has_SSE2() {
    unsigned long ECX, EDX, CPUInfo[4], CPUInfoExt[4];
    info( CPUInfo, CPUInfoExt, ECX, EDX );
    return (EDX_SSE2_bit & EDX) != 0;
  }

  bool
  has_SSE3() {
    unsigned long ECX, EDX, CPUInfo[4], CPUInfoExt[4];
    info( CPUInfo, CPUInfoExt, ECX, EDX );
    return (ECX_SSE3_bit & ECX) != 0;
  }

  bool
  has_SSSE3() {
    unsigned long ECX, EDX, CPUInfo[4], CPUInfoExt[4];
    info( CPUInfo, CPUInfoExt, ECX, EDX );
    return (ECX_SSSE3_bit & ECX) != 0;
  }

  bool
  has_SSE41() {
    unsigned long ECX, EDX, CPUInfo[4], CPUInfoExt[4];
    info( CPUInfo, CPUInfoExt, ECX, EDX );
    return (ECX_SSE41_bit & ECX) != 0;
  }

  bool
  has_SSE42() {
    unsigned long ECX, EDX, CPUInfo[4], CPUInfoExt[4];
    info( CPUInfo, CPUInfoExt, ECX, EDX );
    return (ECX_SSE42_bit & ECX) != 0;
  }

  bool
  has_SSE4a() {
    unsigned long ECX, EDX, CPUInfo[4], CPUInfoExt[4];
    info( CPUInfo, CPUInfoExt, ECX, EDX );
    return (ECX_SSE4A_bit & CPUInfoExt[2]) != 0;
  }

  bool
  has_SSE5() {
    unsigned long ECX, EDX, CPUInfo[4], CPUInfoExt[4];
    info( CPUInfo, CPUInfoExt, ECX, EDX );
    return (ECX_SSE5_bit & CPUInfoExt[2]) != 0;
  }

  bool
  has_3Dnow() {
    unsigned long ECX, EDX, CPUInfo[4], CPUInfoExt[4];
    info( CPUInfo, CPUInfoExt, ECX, EDX );
    return (EDX_3Dnow_bit & CPUInfoExt[3]) != 0;
  }

  bool
  has_3DnowExt() {
    unsigned long ECX, EDX, CPUInfo[4], CPUInfoExt[4];
    info( CPUInfo, CPUInfoExt, ECX, EDX );
    return (EDX_3DnowExt_bit & CPUInfoExt[3]) != 0;
  }

  struct CPUVendorID {
    uint32_t ebx;
    uint32_t edx;
    uint32_t ecx;

    string
    toString() const {
     return string(reinterpret_cast<const char *>(this), 12);
    }
  };

  string
  cpuInfo() {
    unsigned long CPUInfo[4];
    cpuId( CPUInfo, 0 );
    CPUVendorID vendorID;
    vendorID.ebx = uint32_t(CPUInfo[1]);
    vendorID.edx = uint32_t(CPUInfo[3]);
    vendorID.ecx = uint32_t(CPUInfo[2]);

    map<string,string> vendorIdToName;

    vendorIdToName["GenuineIntel"] = "Intel";
    vendorIdToName["AuthenticAMD"] = "AMD";
    vendorIdToName["CyrixInstead"] = "Cyrix";
    vendorIdToName["CentaurHauls"] = "Centaur";
    vendorIdToName["SiS SiS SiS "] = "SiS";
    vendorIdToName["NexGenDriven"] = "NexGen";
    vendorIdToName["GenuineTMx86"] = "Transmeta";
    vendorIdToName["RiseRiseRise"] = "Rise";
    vendorIdToName["UMC UMC UMC "] = "UMC";
    vendorIdToName["Geode by NSC"] = "National Semiconductor";

    string vendorIDString = vendorID.toString();

    map<string,string>::iterator it = vendorIdToName.find(vendorIDString);
    string vendorName = (it == vendorIdToName.end()) ? "Unknown" : it->second;

    return fmt::format(
      "Max instruction ID: {} Vendor ID: {} Vendor name: {}",
      CPUInfo[0], vendorIDString, vendorName
    );
  }
}

#endif
#endif
```

### Quick search

### Table of Contents

- rang - Colors for your Terminal
- Zstr
- C++ API

«
hide menu

menu
sidebar
»

### Navigation

- index
- toc
- Utils »
- Program Listing for File CPUinfo.cc

© Copyright 2021, Enrico Bertolazzi.
Created using Sphinx 4.2.0.
